# Illness cognitions and parental stress symptoms following a child’s cancer diagnosis

**DOI:** 10.3389/fpsyg.2024.1436231

**Published:** 2024-09-24

**Authors:** Inese Lietaviete, Baiba Martinsone

**Affiliations:** Department of Psychology, University of Latvia, Riga, Latvia

**Keywords:** childhood cancer, psychosocial assessment, parental stress, illness cognitions, psycho-oncology

## Abstract

**Objective:**

This research aims to explore parents’ cognitive beliefs, specifically illness cognitions, in response to their children being diagnosed with cancer. This study is an initial step toward providing regular psychosocial assessment as a standard for psychosocial care for children with cancer and their families in Latvia.

**Methods:**

Data were collected from 120 parents (mostly mothers, *n* = 109) as an initial evaluation of the psychosocial risks faced by families participating in the support program “Holistic and Multidisciplinary Support for Children with Functional Disabilities and Their Family Members,” which was managed by the Children’s Hospital Foundation at the Children’s Clinical University Hospital in Riga (Latvia) from 2020 to 2023. The patients comprised 66 boys and 54 girls (M age = 7.1, SD = 4.7, range: 0–17 years) with diverse cancer diagnoses. The parents completed the Latvian version of the Psychosocial Assessment Tool [adapted from PAT 3.1], with five subscales included in the data analysis (Social Support, Child Problems, Family Problems, Stress Reactions, and Family Beliefs). The Family Belief subscale was adjusted specifically to address the unique objectives and research questions of the current study.

**Results:**

Significant correlations were found between children’s psychological problems (e.g., getting upset about medical procedures, hyperactivity, excessive use of electronic devices, etc.) and parental stress reactions after diagnosis and with self-reported symptoms of anxiety and depression. The associations were statistically significant, even after controlling for sociodemographic and medical factors such as diagnosis. The relationship between children’s problems and parental stress reactions was partly mediated by Family Beliefs about illness. The most informative beliefs associated with parental stress symptoms and the family’s psychosocial risk level were identified, and significant results were found according to the main component of beliefs (*catastrophic* vs. *optimistic*) that explained 42% of the variance in the Family Beliefs subscale.

**Implication:**

Understanding the habitual responses to stress and identifying the thinking patterns of parents that lead to distorted views and maladaptive coping are essential for customizing personalized interventions to enhance treatment compliance. The Latvian version of PAT is a useful psychosocial screening measure in pediatric oncology settings.

## 1 Introduction

Parents of children diagnosed with cancer face high levels of situational uncontrollability and uncertainty and have few possibilities to regulate events, but they have the ability to regulate the cognitive appraisal of the situation. Despite childhood cancer being a rare disease, occurring in fewer than 1 in 2,000 cases, it still accounts for around a quarter of all childhood deaths (European Cancer Inequalities Registry, 2023). It is estimated that in 2022, over 14,000 children aged 0 to 19 years were diagnosed with cancer, and 2,100 died from cancer in Europe (European Cancer Inequalities Registry, 2023).

Cancer incidence, mortality rates, and survival percentages vary considerably among EU countries and regions. Overall, the 5-year survival rate for 2010–2014 in Europe for 0-14-year-old children and adolescents was 81%. Results from a population-based study in Europe (Botta et al., 2022) show that lymphomas had the highest 5-year age-adjusted survival rate (over 90%), while CNS tumors had the lowest (around 60%). These cancer incidence and survival percentages are similar in Latvia, where about 50 children are newly diagnosed with cancer every year.

The psychosocial needs and vulnerabilities of children with cancer and their families are well-documented in pediatric cancer literature and include an increased risk of parental distress, posttraumatic stress symptoms (PTSS), and anxiety (Bakula et al., 2019). Children might go through invasive treatments, encounter painful side effects, and be separated from social activities and typical roles, leading to an increased likelihood of experiencing psychological distress and elevations in negative psychosocial outcomes, including internalizing and externalizing (Rodriguez et al., 2012). Previous findings also indicate that pediatric cancer significantly affects parents’ perceptions of self-and family functioning, particularly within the first year following diagnosis (Pai et al., 2007). This period is marked by heightened psychological distress among parents, which gradually decreases over time, but a significant number of parents still experience clinical distress even after 5 years.

Patients who receive a diagnosis of a chronic illness (e.g., cancer) hold their own perceptions and understanding of their condition, which are referred to as illness cognitions. Illness cognitions encompass a patient’s viewpoint, interpretation, and comprehension of the illness and its treatment, as proposed by Leventhal’s common-sense model (CSM) of self-regulation (Leventhal, 1986). This is a widely used theoretical framework that explains how individuals perceive their illness, select coping strategies, and adjust their views based on their coping experiences (Ames et al., 2024; Hagger and Orbell, 2003; Hagger and Orbell, 2022; Horwood et al., 2024; Hagger et al., 2017). Additionally, the effectiveness of these strategies on recovery is context-dependent, aligning with the ‘goodness-of-fit’ hypothesis.

The way parents perceive the stressfulness of their child’s cancer diagnose and its severity, the intensity of treatment, and their own ability to cope with their child’s disease are significantly linked to their level of distress (Kazak et al., 2004). This distress and disorganization are understandable and even expected. Parents fear for their child’s life and face the challenge of making delicate treatment decisions while interacting with numerous unfamiliar healthcare professionals. Additionally, they must adapt to new roles and responsibilities to meet the demands of treatment, all while providing comfort and support to their child.

Recognizing and understanding beliefs is crucial because they can serve as significant predictors or factors that influence the outcome for caregivers and patients. Parents who held beliefs that their child might not survive treatment, could still die from cancer even after treatment ended, or found the cancer journey particularly challenging demonstrated higher levels of PTSS (Kazak et al., 1998). Parents’ beliefs about treatment-related suffering and the side effects of the treatment (such as chemotherapy-induced nausea, pain, and hair loss) were significantly correlated with general distress measures, like anxiety, more symptoms of psychopathology, and greater hopelessness, as well as PTSS, such as intrusive thoughts, arousal, and avoidance (Kazak et al., 2004). Similarly to beliefs about treatment-related suffering, parents’ beliefs that a child was likely to die and that the cancer experience would be upsetting for the family were also linked to higher levels of psychological distress, less adaptive family functioning, and fewer spiritual beliefs among parents. On the other hand, more adaptive family functioning was connected with beliefs about caregivers’ competence and connection with others. Beliefs about connection were also inversely correlated with hopelessness (Kazak et al., 2004).

Resiliency factors that help families to recover include rapid internal mobilization and reorganization, social support from the healthcare team, the extended family, and the workplace, as well as cognitive reframing to make the situation meaningful, understandable, and more manageable (McCubbin et al., 2002). Research shows that optimistic parents who perceive benefits are less distressed compared to those who lack this optimistic outlook (Fotiadou et al., 2008). Additionally, parental distress has an impact on the child’s distress, highlighting the influence of parental beliefs on the psychological adjustment of the entire family. In a meta-analysis of 28 research works, a statistically significant association was found between overall parent and child distress (*r* = 0.32, *p* < 0.001), such that increased parent-reported distress was associated with increased distress in their children (Bakula et al., 2019). Therefore, gaining better insight into how parents perceive their child’s illness could lead to a greater understanding of maladaptive adjustment within the family.

Although illness cognitions have significant implications for adjustments to childhood cancer in families, the strength of this relationship among family members (mothers vs. fathers, grandparents, siblings, etc.) remains understudied. Of the instruments designed to measure illness perceptions in members of families with children with cancer, the most frequently used are the Family Illness Beliefs Inventory (FIBI) (Kazak et al., 2004) and the Illness Cognition Questionnaire – Parent Version (Sint Nicolaas et al., 2016). The Psychosocial Assessment Tool (PAT 3.1) (Kazak et al., 2018) also has the 10-item subscale Family Beliefs. However, there is still a need for more comprehensive explorations of how children are affected by their parents’ illness perceptions and how it influences the children’s own perspectives. Additionally, there is a notable lack of emphasis on assessing illness perceptions among siblings of pediatric cancer patients. Lastly, there is a need for more research into the changes in illness cognitions during long-term adjustment to disease and their predictive contribution to adjustment processes in both patient and parent groups (Sint Nicolaas et al., 2016).

Another important aspect that requires careful consideration is the cultural relevance of the measures used to assess cognitive beliefs about illness. The need to understand the cultural beliefs regarding the causes of cancer in any given population arises from the fact that understanding illness and behavioral responses to illness are basic factors influencing coping in such cases. The purpose of this study is to assess illness cognitions and psychosocial risks of parents of children diagnosed with cancer using the Latvian version of the Psychosocial Assessment Tool (based on PAT 3.1). The method has been adjusted to the Latvian cultural context and customized to address the unique objectives and research questions of the current study while ensuring that the data collected are directly relevant to the study’s focus. To date, no comprehensive studies on the illness cognitions and psychosocial adjustment of parents of children with cancer in Latvia have been conducted.

Based on the theoretical framework and the results of previous studies, the research questions for this study are:

1. What are the main dimensions of the cancer-related cognitions of parents after their child has been diagnosed with cancer?
2. Which beliefs may be most informative about parental stress symptoms and the family’s psychosocial risk level?
3. What is the relation between the cancer-related cognitions held by parents and perceived support from their healthcare team, extended family, and other social systems?
4. To what extent do cancer-related cognitions mediate the relationship between a child’s psychological problems and parental distress symptoms after diagnosis?
5. What is the ability of the Latvian version of the Psychosocial Assessment Tool to identify families at clinical risk?

## 2 Materials and methods

### 2.1 Participants

Data were collected as an initial assessment of the psychosocial risks of families included in the psychosocial support program “Holistic and Multidisciplinary Support for Children with Functional Disabilities and Their Family Members” (Lietaviete, 2023), which was managed by the Children’s Hospital Foundation and the European Social Fund at the Children’s Clinical University Hospital in Riga (Latvia) from 2020 to 2023. The inclusion requirements were being the parent/caregiver of a child ages birth through 17 years with newly diagnosed cancers or a recurrence of a primary tumor that required chemotherapy and/or radiation. There were no specific exclusion criteria related to the disease; however, patients referred directly to palliative care at the time of diagnosis (with a prognosis of less than 2 months to live) and those who were not fluent in Latvian were ineligible.

Assessments were collected from 120 parents (mostly mothers, *n* = 109; 91%) with children newly diagnosed with cancer. In the cases of two families, a grandmother functioned as a parent. The participants were between 25–68 years old (mothers: M age = 40.94, SD = 6.83, fathers: M age = 39.45, SD = 3.64). The majority of participating families included two parents (*n* = 102; 85%); however, data were obtained from the main caregiver, i.e., the mother or father who was with the child at the hospital. The gender disparity mirrors the actual circumstances in pediatric cancer care; however, due to this imbalance, gender was excluded as a variable in the subsequent analysis.

The childhood cancer diagnoses in the sample were leukemia and lymphoma (*n* = 74; 62%), brain and spinal cord tumors (*n* = 19; 16%), solid tumors, e.g., sarcoma (*n* = 23; 19%), and others (*n* = 4; 3%). There were 66 boys and 54 girls among the patients (M age = 7.13, SD = 4.73, range: 0–17 years). Additional information can be found in Table 1.

**Table 1:** Sociodemographic characteristics of participating parents (*n* = 120) and medical characteristics of their children.

	% (*n*) or mean (SD)		Missing cases
Parents’ age (years)	40.8	(6.6)	3
**Sex**			
Male Female	9% 91%	(11) (109)	
**Family structure**			
Married/Partnered Single parent Other	85% 13% 2%	(102) (16) (2)	
Number of children 1 2 3 >3	2.3 23% 40% 26% 11%	(1.1) (27) (47) (31) (14)	1
**Child medical characteristics**			
Age at diagnosis (years) 0–4 5–9 10–14 14–17	7.1 40% 29% 20% 11%	(4.7) (48) (35) (24) (13)	
**Primary childhood cancer diagnosis**			
Hematological cancer CNS tumor Solid tumor Other	62% 16% 19% 3%	(74) (19) (23) (4)	
Recurrence of primary tumor	8%	10	
**Intensity of treatment**			
Level I Level II Level III Level IV	3% 30% 43% 24%	(4) (36) (51) (29)	

### 2.2 Measures

The Latvian version of the Psychosocial Assessment Tool adapted from PAT 2.0 (Pai et al., 2008) and PAT 3.1 (Kazak et al., 2018) was used to collect information about parental beliefs and stress reactions after diagnosis. The PAT is a screening tool widely used to identify family psychosocial risk in families with children diagnosed with cancer. Based on social-ecological theory and the Pediatric Psychosocial Preventative Health Model (PPPHM) (Kazak, 2006), it evaluates psychosocial risks across the child’s social environment on seven subscales: Family–Structure/Resources, Social Support, Child Problems, Sibling Problems, Family Problems, Stress Reactions, and Family Beliefs. Five subscales were analyzed in the Latvian version: Social Support, Child Problems, Family Problems, Stress Reactions, and Family Beliefs.

In a similar way to PAT 3.1, the responses to items had variable formats (multiple choice, 4-point Likert scale, Yes/No). Each scale was scored by summing the number of positively scored items and dividing the sum by the number of items in that scale, resulting in a score that ranges from 0.00 to 1.00. Two binary items from the Family Problems subscale, related to symptoms of depression and anxiety, were analyzed as individual measures for self-reported symptoms of depression and anxiety and included in further data analysis.

Parental beliefs about a child’s cancer are most frequently evaluated through specific questionnaires, e.g., the Family Illness Beliefs Inventory (FIBI, 41 items) (Kazak et al., 2004) and the Illness Cognition Questionnaire – Parent Version (18 items) (Sint Nicolaas et al., 2016). Another approach to measuring parental beliefs is to include some selected items from the FIBI in broader self-report scales like the PAT. The initial version (Kazak et al., 2001) was modified in PAT 2.0 (Pai et al., 2008; Kazak et al., 2015) and later in PAT 3.1 (Kazak et al., 2018). As a PAT subscale, Family Beliefs highly relate to psychosocial risk, but beliefs are difficult to measure and may not necessarily relate consistently to other constructs. For example, items assessing beliefs related to treatment-related suffering and death from the Family Illness Beliefs Inventory (Kazak et al., 2004), included on the PAT 2.0 form (Pai et al., 2008), were not included in the Family Beliefs subscale score due to poor reliability. Nevertheless, these beliefs can provide substantial clinical utility and offer valuable insights into the family’s psychosocial risk level. Due to the multidimensional nature of the Family Beliefs subscale, only four items from PAT 2.0 were analyzed. The internal consistency of these four items were *α* = 0.59 (Pai et al., 2008).

The current research began with a 10-item version adapted from the PAT 2.0 Family Belief subscale. This scale included both positively framed and strongly negative items, aiming to provide a comprehensive representation of parental beliefs regarding their child’s illness and to capture a broader spectrum of responses and nuances in attitudes. However, the initial reliability of the subscale was low, with an alpha coefficient of 0.47. By excluding four items that had ambiguous phrasing (e.g., *“Everything happens for a reason”*) or demonstrated low internal correlation with other items in the subscale, the internal consistency of the remaining six items improved to *α* = 0.58. These six items included in the Family Beliefs subscale score are marked with asterisks. The psychometric properties of all subscales, along with their comparison to PAT 2.0 and PAT 3.1, are presented in Table 2.

**Table 2:** Descriptive statistics and internal consistency (*n* = 120) of each scale and composite scale of the Latvian version of the PAT and comparison from the literature on PAT 2.0 and PAT 3.1.

Subscales	*M*	SD	Cronbach’s *α*	PAT 2.0(Pai et al., 2008) Cronbach’s *α*	PAT 3.1 (Kazak et al., 2004) KR 20
**PAT**					
Social support Child problems Family problems Stress reactions Family Beliefs PAT total	0.46 0.40 0.27 0.60 0.41 1.72	0.17 0.09 0.21 0.15 0.12 0.41	0.84 0.65 0.60 0.71 0.58 0.82	0.69 0.81 0.72 0.64 0.59 0.81	0.59 0.80 0.64 0.84 0.59 0.81

KR20, the Kuder–Richardson 20 coefficient.

### 2.3 Procedure

Data on cancer-related beliefs and stress reactions of the parents of a child diagnosed with cancer were collected in two separate studies. Sample 1 consisted of parents who were included in the psychosocial support program “Holistic and Multidisciplinary Support for Children with Functional Disabilities and Their Family Members” in 2020–2021, and sample 2 consisted of parents who participated in the same program in 2022–2023. Both studies were conducted at the Children’s Clinical University Hospital in Riga, which serves as the sole center for pediatric oncology in Latvia. The studies were similar in terms of including the parents of a child diagnosed with cancer and the time since diagnosis, which ranged from 1 to 12 months (*M* = 4.66, SD = 2.29).

Participants completed a paper-and-pencil Latvian version of the PAT. The measure evaluated families’ psychosocial risks across seven domains, but only five are included in this analysis due to the constructs’ clarity: Social Support, Child Problems, Family Problems, Stress Reactions, and Family Beliefs.

### 2.4 Data analysis

After removing data from three families where responses were missing for >50% of the items, surveys of the primary caregiver from 120 families were analyzed. Data analyses were carried out on R version 4.20 using the *psych* package (Revelle, 2023). The study was designed cross-sectionally. A series of correlational and regression mediation analyses were performed, adjusted with BH methods. The choice between tests was based on the nature of the data, specifically the assumptions of normality (Shapiro–Wilk test for normality was conducted). A *p* value <0.05 was considered to be statistically significant for all tests.

Descriptive statistics were used to describe the sociodemographic variables of participants (age, sex, marital status, number of children in family) and patients (age, sex, primary childhood cancer diagnosis). The subscales’ scores were weighted by the number of items in the subscale, and the total score was the sum of four subscales (the Family Belief subscale was excluded – see below). Descriptive statistics (mean, standard deviation, Cronbach’s alpha) were used to describe the subscale results (Table 2).

Principal component analysis (PCA) was employed to analyze survey responses regarding psychological aspects related to children’s diagnosis. Many psychological variables were intercorrelated, and a multivariable regression model with these variables might result in over-parameterization. The components in PCA are arranged in such a way that the first component captures the highest variance present in the data. Each subsequent component captures the next highest variance in the direction orthogonal to the first component, and so on. PCA was used to identify the latent structure of the 10-item Family Beliefs subscale and the 13-item Stress Reactions subscale. Component loadings of 0.30 or higher were considered significant based on the sample size of 120. In order to examine the relationship between the Family Beliefs and Stress Reactions subscales’ main components, Pearson’s product–moment correlation was calculated.

Pearson’s correlations were used to examine the relationship between risk area-specific subscales (Social Support, Child Problems, Family Problems, Stress Reactions), beliefs about illness, and parents’ age. Welch two-sample t-tests were performed to check the significance of the difference between groups with and without depression and anxiety according to Child Problems and Stress Reactions. Effect sizes were labeled following Cohen’s recommendations (cited in Ben-Shachar et al., 2020).

Spearman’s rank correlations were used to examine the relationship between beliefs and parental stress symptoms after diagnosis, self-reported anxiety, and depression symptoms in family and social support networks. The Wilcoxon rank sum test with continuity correction was employed to check the differences in beliefs between the targeted (higher) psychosocial risk group (PAT total score ≥ 2.5) and the universal group of parents (PAT total score ≤ 2.5).

Causal mediation analysis with multiple linear regression was conducted to examine the impact of Family Beliefs on the connection between a child’s problems and parental stress reactions following a diagnosis. The non-parametric bootstrap procedure (samples = 5,000; 95% bias-corrected confidence intervals) was employed to estimate the indirect and direct effects and ascertain the associated standard errors and significance levels.

## 3 Results

### 3.1 Descriptive statistics

The means, standard deviations, and Cronbach’s alphas for the Latvian version of the PAT subscales used in the study are reported in Table 3. All of the subscales showed nearly acceptable to good Cronbach’s alphas (0.60–0.84) except for Family Beliefs, which exhibited lower internal consistency (0.58) than the other subscales, probably due to assessing a broader, multi-dimensional concept.

**Table 3:** Loading matrix with eigenvalues for PC1 and PC2 beliefs.

Family Beliefs	PC1	PC2
Cancer is a death sentence.* My child will be in a lot of pain.* We can make good treatment decisions.* This is a disaster. Our family will be closer because of this.* People will pull away from us. Our marriage or family will fall apart. We’re going to beat this.* The doctors will know what to do.* Everything happens for a reason.	**−0.512** **−0.302** **0.450** −0.272 0.249 −0.130 −0.218 **0.358** 0.**334** −0.065	−0.103 0.153 0.191 **−0.521** **−0.466** 0.234 0.225 **−0.383** **0.345** 0.266

*Items included into Family Beliefs subscale score. With bold - Component loadings of 0.30 or higher were considered significant.

Our findings are consistent with those presented by Kazak et al. (2018) regarding the validation of PAT 3.1 and its earlier iterations. For instance, in PAT 2.0, the internal consistency of the Family Beliefs subscale was *α* = 0.59, with only 4 out of 10 items being analyzed (Pai et al., 2008). In PAT 3.1, the internal consistency for the Family Belief scale was also 0.59, which is considered adequate but less internally consistent than other subscales (Kazak et al., 2018).

**Figure 1:**
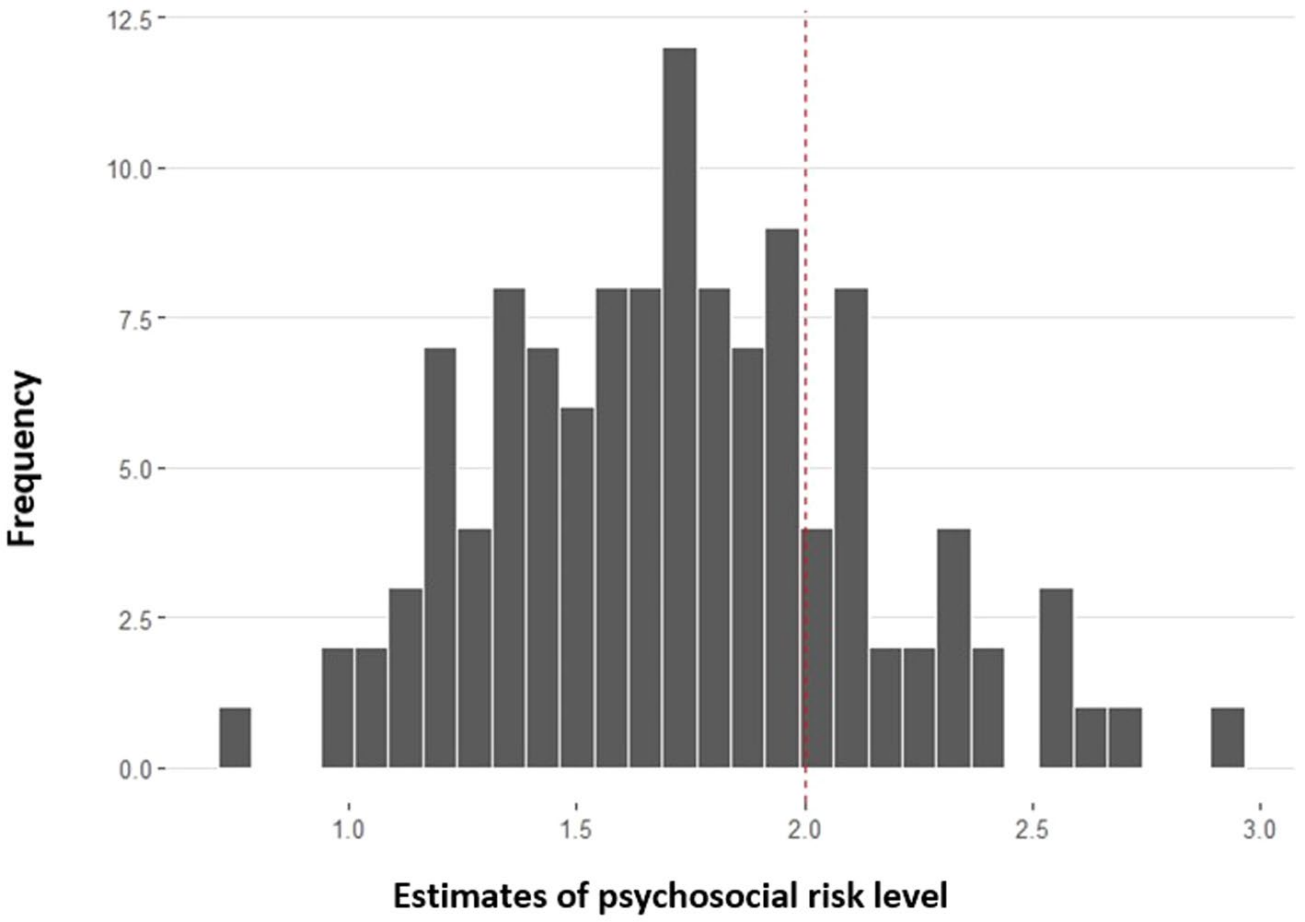
Distribution of families’ psychosocial risk levels after child’s cancer diagnosis (*n* = 120).

The total score of the Latvian version of the PAT was compiled as a sum of four risk area-specific subscales (Social Support, Child Problems, Family Problems, Stress Reactions). The Family Belief subscale was not included in the total score because of its low internal consistency. Cutoff scores were used to classify families into the three risk levels proposed by the PPPHM, based on both theoretical foundations and prior empirical analyses, as outlined by Kazak (2006): total scores <2.00 are Universal, between 2.00 and 2.50 are Targeted, and over 2.50 are Clinical (Figure 1).

In this study, 77.5% of the sample scored at the Universal level, and 22.5% were in the Targeted range (of which 5% were in the Clinical tier).

### 3.2 Principal component analysis of subscales

To answer the research question concerning the main dimensions of parents’ cancer-related cognitions after their child has been diagnosed with cancer, PCA was employed to analyze the structure of the 10-item Family Beliefs and 13-item Stress Reactions subscale. PCA was selected as a technique to extract dominant patterns from a dataset, reduce its dimensionality, and provide a clearer understanding of the data’s inherent variability.

**Figure 2:**
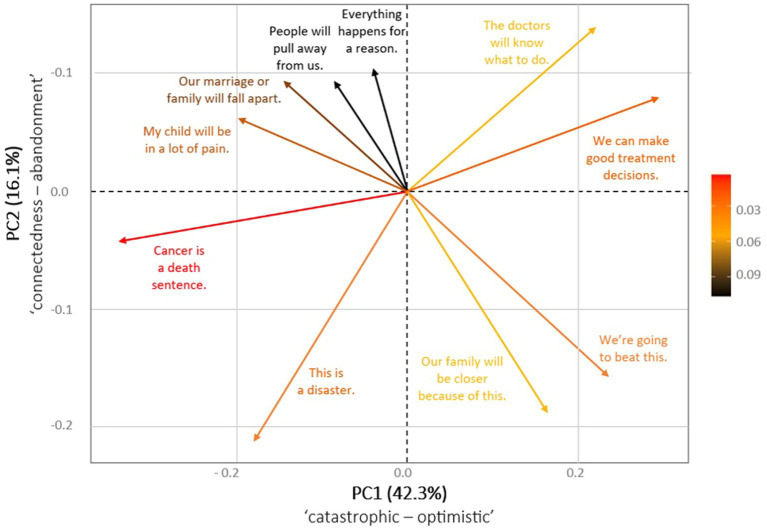
Biplot of the cognitions with respect to the principal components of Family Beliefs.

The PCA analysis revealed two distinct component eigenvectors, PC1 and PC2, which explained 42 and 16% of the variance in the Family Beliefs subscale. PC1 largely reflected an optimistic thinking pattern (*“We can make good treatment decisions”*), supplemented in the opposite direction by a catastrophic thinking pattern (*“Cancer is a death sentence”*). PC2 reflected changes in connectedness with others (*“Our family will be closer because of this”*) and, in the opposite direction, fear of abandonment (*“Our marriage or family will fall apart”; “People will pull away from us”*). The data are not sufficiently clear to warrant further analysis of PC2; therefore, only the PC1 dimension (*catastrophic* vs. *optimistic*), which explains 42% of the variance, was used for further analysis. The full loading matrix containing the eigenvalues for PC1 and PC2 and the dimensional representations for PC1 and PC2 can be found in Table 3 and Figure 2, respectively.

The PCA analysis also revealed two distinct components in the stress reactions of parents after diagnosis: PC1 and PC2 explained 27 and 19% of the variance in the Stress Reactions subscale, respectively. PC1 (*individual discomfort*) largely reflected the personal emotional discomfort of parents, supplemented in the opposite direction by problems in their professional career due to their child’s illness. PC2 (*changes in parental role*) reflected changes in the parental role externally (e.g., medical care, difficulties in setting boundaries on the child’s behavior, etc.) and, in the opposite direction, internally (e.g., bad dreams about their child’s cancer). The full loading matrix for PC1 and PC2 can be found in Table 4.

**Table 4:** Loading matrix with eigenvalues for PC1 and PC2 stress reactions.

Stress Reactions	PC1	PC2
Difficulties in accepting changes in professional life Existential questions about the reasons for the child’s illness Intense discomfort during the child’s medical procedures Discomfort (sweating, feeling angry) when asked about the child’s diagnosis Severe mood swings Difficulties in relaxing due to increased stress (muscle tension, pain, sleep) Lack of time for self-care (negative changes in eating habits, lack of exercise) Bad dreams or nightmares about the child’s illness Difficulties in ensuring healthy nutrition for the child Changes in parental role (e.g., caring for other family members, household) Difficulties in setting boundaries on the child’s behavior Difficulties in accepting the visual changes and instability of the child’s condition Difficulties in talking to the child about the diagnosis	**−0.501** **0.400** **0.372** **0.307** 0.298 0.275 0.187 0.057 −0.236 0.013 0.001 0.265 0.165	−0.069 0.201 −0.093 0.024 −0.093 −0.130 −0.033 **0.548** **−0.400** **−0.359** **−0.354** **−0.341** −0.295

With bold - Component loadings of 0.30 or higher were considered significant.

### 3.3 Correlation analysis

Pearson’s correlations were used to explore the association between risk area-specific subscales (Social Support, Child Problems, Family Problems, Stress Reactions), beliefs about the child’s illness, and parents’ age (Table 5).

**Table 5:** Pearson’s correlations between subscales and parents’ age (*n* = 120).

Subscales	1	2	3	4	5
1. Social support 2. Child problems 3. Family problems 4. Stress reactions 5. Parental beliefs 6. Age	— ns ns ns ns 0.20*	— 0.31** 0.58** 0.20* ns	— 0.42** 0.32** 0.22*	— 0.35** 0.21*	— Ns

*p < 0.05; **p < 0.01; ns, not significant.

**Figure 3:**
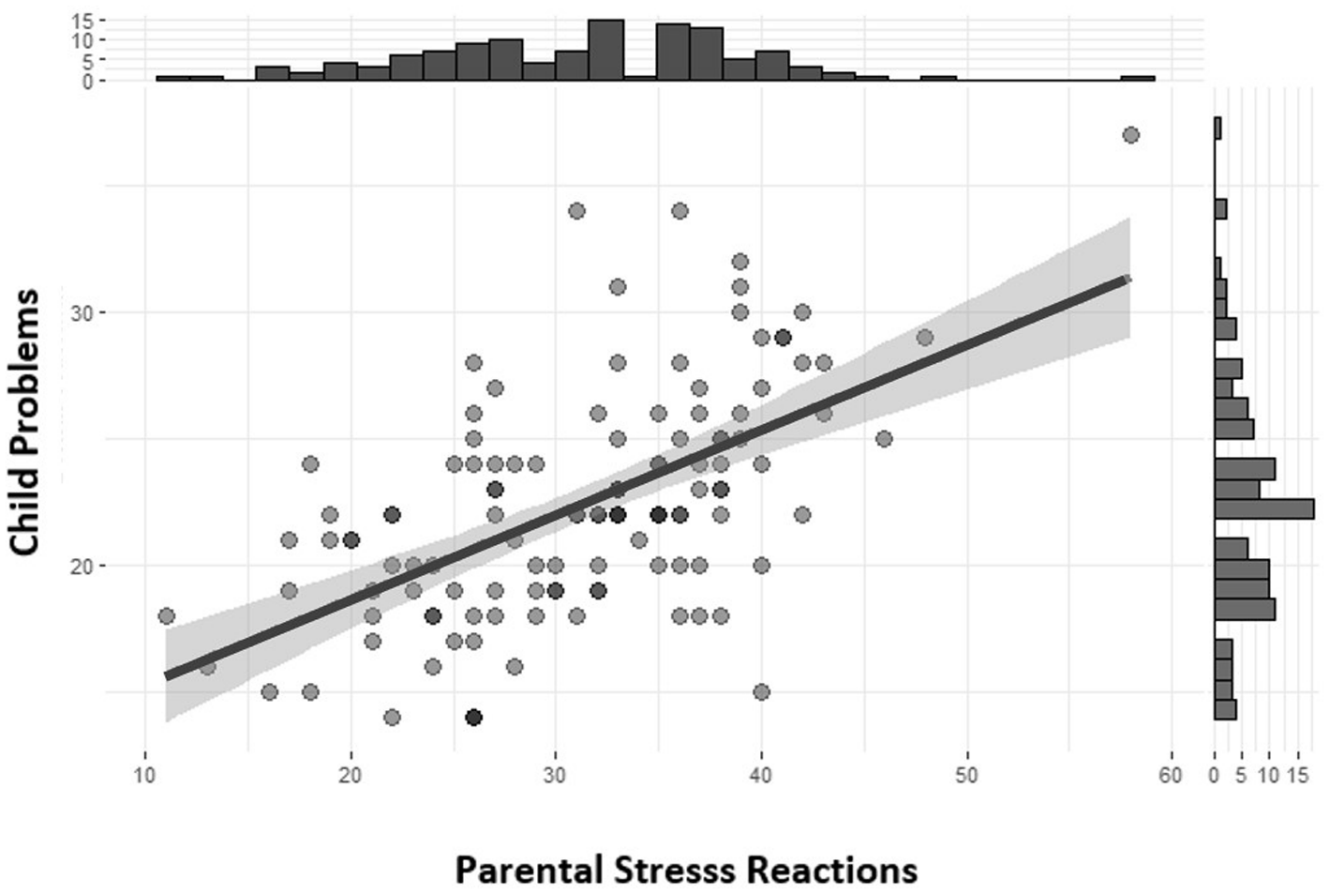
Pearson correlations between a child’s psychological problems and parental stress.

The Pearson correlations presented in Figure 3 show a significant relationship [*r* = 0.58, 95% CI (0.45, 0.69), t (118) = 7.77, *p* < 0.001] between the Child Problems subscale (e.g., getting upset about medical procedures, hyperactivity, worrying, learning problems, having problems making and keeping friends, excessive use of electronic devices, etc.) and the Stress Reactions subscale, which describes parental stress reactions after diagnosis (e.g., mood disturbances, upsetting thoughts, memories or bad dreams about the child’s illness, difficulties in speaking about it with the child and others, etc.). The effect size is very large and is labeled following Funder and Ozer’s (2019) recommendations.

The regression analysis revealed that the association between a child’s problems and parental stress reactions remained statistically significant even after controlling for potential confounding variables. These variables included medical factors such as diagnosis and treatment intensity and sociodemographic factors like the patients’ and parents’ age, marital status, and the number of children in the family. While controlling the child’s problems level, parental stress was found to be higher in families with two or three children compared to those with only one child. In the regression model, while controlling the diagnosis, only solid tumor diagnoses had a significant impact on parental stress reactions (*β* = 0.43, *p* = 0.036); other types of diagnoses had non-significant effects. The impact can be attributed to the malignant nature of sarcomas, which constituted more than half of the solid tumor cases in this diagnostic group (13 out of 23 cases).

Children’s psychological problems also significantly correlate with parents’ mental health problems like self-reported anxiety (binary item in the Family Problem subscale). The Welch two-sample *t*-test testing distributing children’s problems into two groups, with and without parental anxiety (mean in group 1 = 20.66, mean in group 2 = 23.37), suggests that the effect is statistically significant and medium-sized [difference = −2.71, 95% CI (−4.32, −1.09), *t* (94.08) = −3.33, *p* = 0.001; Cohen’s *d* = −0.69, 95% CI (−1.10, −0.27)]. Distributing children’s psychological problems into two more groups, parents with and without self-reported depression, did not yield significant results.

There was also a statistically significant, large effect size distribution of Stress Reactions into two groups, parents with and without self-reported anxiety [difference = −7.25, 95% CI (−10.02, −4.48), *t* (79.99) = −5.21, *p* < 0.001; Cohen’s *d* = −1.17, 95% CI (−1.64, −0.69)], as well as a significant, large effect size distribution of Stress Reactions into two groups, parents with and without self-reported depression [difference = −5.65, 95% CI (−8.38, −2.92), *t* (103.70) = −4.11, *p* < 0.001; Cohen’s *d* = −0.81, 95% CI (−1.21, −0.40)].

**Figure 4:**
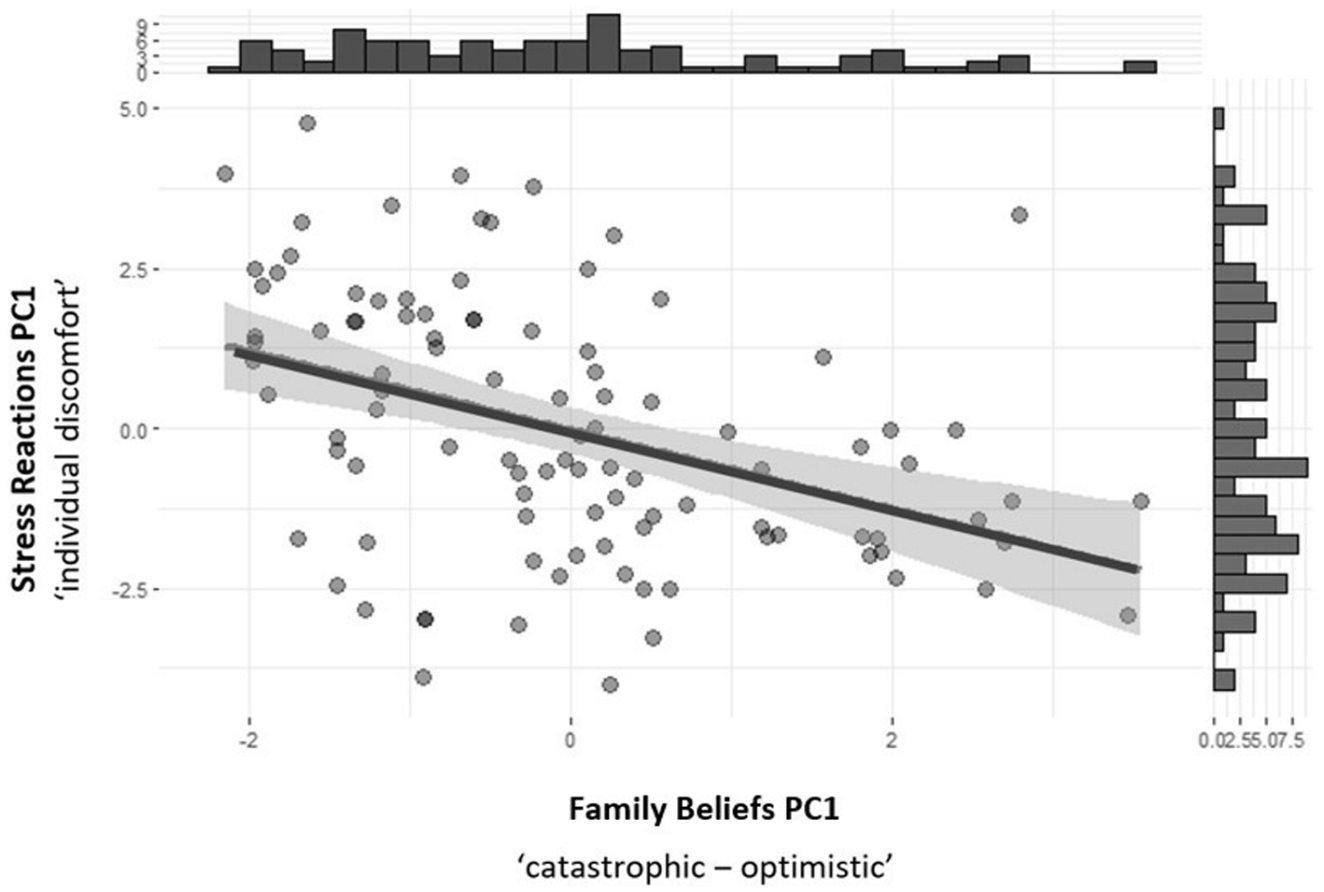
Pearson correlations between main components of Family Beliefs and stress reactions.

The Pearson’s product–moment correlation between Family Beliefs PC1 (*catastrophic* vs. *optimistic*) and Stress Reactions PC1 (*individual discomfort*) was negative, statistically significant, and very large [*r* = −0.42, 95% CI (−0.57, −0.24), *t* (99) = −4.55, *p* < 0.001]. The relationship is presented in Figure 4. No other significant correlations were found between components.

Spearman’s rank correlations were used to answer the research question regarding which beliefs may be most informative about the parental stress symptoms after diagnosis and self-reported anxiety and depression symptoms in the family. The most informative beliefs are presented in Table 6. The effect size ranges from small (rho = 0.18) to medium (rho = 0.34).

**Table 6:** Spearman’s rank correlation between parental beliefs and stress reactions after diagnosis (*n* = 120).

Family beliefs	Self-reported symptoms of anxiety	Self-reported symptoms of depression	Stress reactions after diagnosis
Our marriage or family will fall apart. People will pull away from us. Cancer is a death sentence. This is a disaster.	0.23* 0.18* 0.25** ns	0.18* 0.20* 0.22* ns	0.28* 0.26* 0.34** 0.32**

*p < 0.05; **p < 0.01; ns, not significant.

The belief *“Our family will be closer because of this”* was found to be the most informative about parents’ positive adaptation. The Spearman’s rank correlation rho between this belief and the Family Problems (e.g., anxiety, depression) subscale was negative, statistically significant, and medium (rho = −0.22, S = 2.93e+05, *p* = 0.020).

We did not find a correlation between cancer-related cognitions held by parents and their perceived support from the healthcare team, extended family, and other social systems. The only exception was the belief that *“My child will be in a lot of pain,”* which correlated negatively with perceived support with child care/parenting. The relationship was statistically significant and medium-sized (rho = −0.22, *S* = 3.00e+05, *p* = 0.020). There was a small positive correlation (Pearson’s *r* = 0.20, *p* = 0.030) between social support and parents’ age (Table 3).

To answer the research question regarding which beliefs may be most informative about the family’s psychosocial risk level, the Wilcoxon rank sum test with continuity correction was employed to check the differences in Family Beliefs between the Targeted range group (PAT total score ≥ 2.5) and the Universal group of parents (PAT total score ≤ 2.5). A statistically significant difference was observed between the groups in relation to two beliefs: *“People will pull away from us”* (*W* = 985.5, *p* = 0.009) and *“Cancer is a death sentence”* (*W* = 989, *p* = 0.047).

### 3.4 Mediation tests

Multiple linear regression was employed to answer the research question concerning the extent to which cancer-related cognitions mediate the relationship between a child’s psychological problems and parental distress symptoms after diagnosis. Table 7 shows the impact of Child Problems and Family Beliefs on Stress Reactions. Standardized parameters were obtained by fitting the model on a standardized version of the dataset. 95% Confidence Intervals (CIs) and *p*-values were computed using a Wald *t*-distribution approximation.

**Table 7:** Regression analysis for mediation of Family Beliefs and Child Problems on Stress Reactions (*n* = 120).

Variable	*B*	95% CI	SE *B*	*β*	*R* ^2^	Adj. *R*^2^
**Step 1 (Total effect)**						
Intercept Child problems	0.20*** 0.99***	[0.09, 0.31] [0.72, 1.25]	0.06 0.14	0.56***	0.31	0.30***
**Step 2 (Beliefs model)**						
Intercept Child problems	0.31*** 0.25*	[0.21, 0.41] [0.01, 0.49]	0.05 0.12	0.19*	0.03	0.03*
**Step 3 (Full model)**						
Intercept Child problems Family beliefs	0.10 0.90*** 0.34***	[−0.02, 0.22] [0.64, 1.16] [0.15, 0.53]	0.06 0.13 0.10	0.51*** 0.26***	0.37	0.36***

*p < 0.05; **p < 0.01; ***p < 0.001; CI, confidence interval.

In Step 1, the *R*^2^ value of 0.31 reveals that Child Problems explained 31% of the variance in Stress Reactions [*F* (1, 118) = 52.76, *p* < 0.001]. In Step 2, we fitted a linear model (estimated using OLS) to predict Family Beliefs with Child Problems. The model explained a statistically significant and weak proportion of the variance (*R*^2^ = 0.03) [*F* (1, 118) = 4.23, *p* = 0.042]. In Step 3, the *R*^2^ value of 0.37 reveals that Child Problems and Family Beliefs explained 37% of the variance in Stress Reactions [*F* (2, 117) = 34.94, *p* < 0.001].

The findings reveal that Child Problems (*β* = 0.51, *p* < 0.001) and Family Beliefs positively predicted Stress Reactions (*β* = 0.26, *p* < 0.001). The regression weights for Child Problems subsequently reduced from Model 1 to Model 3 (from 0.56 to 0.51) but remained significant, confirming the partial mediation.

**Figure 5:**
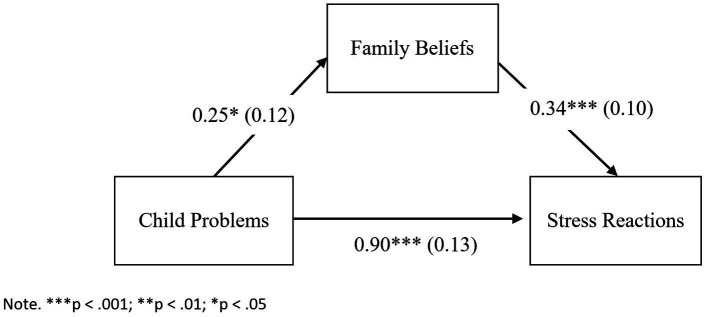
Mediation model for the relationship between child problems and stress reactions as mediated by family beliefs.

Furthermore, Child Problems had direct as well as indirect effects on Stress Reactions. As Figure 5 illustrates, the causal mediation analysis dissected the total effects (0.99) into direct effects (ADE = 0.90, *p* < 0.001) and indirect effects (ACME = 0.09, *p* = 0.02).

Hence, Family Beliefs is a mediator that explains 9% of the variance in the relationship between Child Problems and Stress Reactions.

## 4 Discussion and implications

This study aimed to assess the illness cognitions and psychosocial risks of parents with children diagnosed with cancer using the Latvian version of the Psychosocial Assessment Tool, customized for the local cultural context. This adjustment represents an initial step toward establishing regular psychosocial assessments as a standard in psychosocial care for these families in Latvia.

The study leads to several findings. First, the Latvian version of the PAT demonstrates sufficient reliability and internal consistency to screen families’ psychosocial risk levels. However, further research is needed to examine the scale’s validity and other psychometric properties. Second, the main component of the cancer-related beliefs that explains almost half of the sample’s variance is the *catastrophic* vs. *optimistic* pattern of cognitions. The most catastrophic beliefs are the most informative about parents’ stress adaptation. Third, there is strong evidence of a large effect size between children’s psychological problems and parental stress after the diagnosis, even after controlling for the child’s medical condition and other sociodemographic factors. Fourth, the associations between children’s problems and parental stress reactions are partly mediated by beliefs. These results are in line with the functional approach to illness cognitions within the common-sense model (CSM) of self-regulation (Leventhal et al., 2011).

### 4.1 Initial testing of the Latvian version of the PAT

Understanding the illness cognitions associated with a cancer diagnosis within a specific cultural context is crucial, as these beliefs have a significant impact on individuals’ perceptions of and responses to the illness. The Latvian version of the PAT was modified to suit the Latvian cultural context and tailored to meet the specific objectives and research inquiries of the present study. The psychosocial risk factors were compiled into four risk area-specific subscales: Social Support, Child Problems, Family Problems, and Stress Reactions. These subscales’ internal consistency ranged from acceptable to good (with alpha coefficients ranging from (0.60 to 0.84).

Due to the multi-dimensional nature of the Family Beliefs subscale, its reliability was low. However, that was expected based on the psychometric properties of PAT 2.0 (Pai et al., 2008) and PAT 3.1 (Kazak et al., 2018). Although the Family Belief subscale was omitted from the total score due to its low internal consistency, the items within the subscale yielded valuable insights and predicted certain facets of parental stress reactions following the diagnosis.

Families were classified into three risk levels based on their total score: 77.5% of the sample scored at the Universal level (comprising capable and adaptable families facing health-related stressors), 22.5% were in the Targeted range (a smaller group that faced a heightened risk of ongoing psychosocial challenges), and 5% of the latter group were in the Clinical tier (families exhibiting more evident symptomatology). Although the scoring system of the Latvian version differs from PAT 3.1 due to the reduced number of scales, the distribution of psychosocial risk levels in the sample is comparable to the proportions suggested by the PPPHM and the PAT 3.1 validation sample (62.5% at the Universal level, 36.9% in the Targeted range).

Overall, this study has demonstrated that the Latvian version of the PAT is internally consistent instrument for screening stress reactions and psychosocial risk levels in families with children newly diagnosed with cancer. However, the instrument’s validity requires further examination in future research to gain a deeper understanding of the cultural factors influencing cancer-related beliefs and stress reactions among parents in Latvia.

### 4.2 Catastrophic vs. optimistic illness cognitions in relation to stress

PCA was employed to examine the structure of the Family Beliefs and Stress Reactions subscales to better understand the interrelationships between cognitions and stress. PCA revealed two distinct component eigenvectors in the Family Beliefs subscale, PC1 and PC2, which explained 42 and 16% of the variance, respectively. PC1 reflected *catastrophic* vs. *optimistic* thinking patterns, while PC2 reflected changes in *connectedness* with others and fear of abandonment. Similarly, two principal components explained 27 and 19% of the variance in the Stress Reactions subscale: PC1 reflected *individual discomfort* caused by the child’s illness and changes in professional life, while PC2 reflected changes in *parental roles*. Pearson’s product–moment correlation between Family Beliefs PC1 (*catastrophic* vs. *optimistic thinking*) and Stress Reactions PC1 (*individual discomfort*) exhibited a negative, statistically significant, and very large correlation.

The results reflect the primary connections between cognitions and stress, aligning with findings from other studies where heightened distress was associated with parental illness cognitions centered on feelings of helplessness and non-acceptance of illness (Sint Nicolaas et al., 2016; Bilani et al., 2019) or treatment-related suffering (Kazak et al., 2004). Moreover, this study provides valuable insights into the structure of parents’ cancer-related cognitions by identifying the main components (directions) in which the data vary the most.

The *catastrophic* vs. *optimistic* component of cognitions captures a substantial portion of the overall data variance and may represent optimism as a primary catalyst for change. Furthermore, optimism is not just a characteristic of an adaptive thinking pattern but can be seen as a fundamental personal disposition and personality trait. Therefore, our findings are in line with studies where dispositional optimism, negative affect, and neuroticism were consistently predictive of stress trajectories in both children and parents (Sharp et al., 2022).

The strength of the dispositional predictors in contrast to the limited influence of medical factors, as presented in some studies (Sharp et al., 2022; Phipps et al., 2009), highlights the importance of attention to premorbid personality variables when identifying individuals at the highest risk of adjustment difficulties in the pediatric cancer context. In this regard, our findings indicate the potential for early screening for dispositional optimism/pessimism by assessing thinking patterns. The items with the highest loadings that constitute the *catastrophic* vs. *optimistic thinking* component (e.g., *“Cancer is a death sentence”* vs. *“We are capable of making sound treatment decisions”*) could therefore be very informative about personality dispositions and predict psychological outcomes after treatment.

### 4.3 Children’s psychological problems and parental stress after diagnosis

The study found a significant correlation with a very large effect size between a child’s psychological problems (e.g., getting upset about medical procedures, hyperactivity, excessive use of electronic devices, etc.) and their parents’ acute stress reactions after diagnosis (e.g., upsetting thoughts, memories or bad dreams about the child’s illness, mood disturbances, difficulties in speaking about cancer diagnosis with the child and with others, etc.). The findings are in line with other studies summarized in a meta-analytical review (Bakula et al., 2019) that shows a significant association between parent and child distress (*r* = 0.32, *p* < 0.001).

However, the strength of the relationship (Pearson’s *r* = 0.58, *p* < 0.001) was surprisingly high in comparison with similar studies (Kazak et al., 2018) using PAT 3.1, where the association between Child Problems and Stress Reactions was significant but low (*r* = 0.20, *p* < 0.01). The variation in effect size can be attributed to differences in subscale design (the Latvian version of the Stress Reactions subscale represents a broader spectrum of parents’ stress adaptation reactions, using 14 items instead of the four items used in PAT 3.1). The disparity of effect size may also be explained by cultural influences, sample disparities, response bias (distressed parents report more child distress), and other underlying factors.

Moreover, the study found significant correlations between a child’s psychological problems and their parents’ mental health problems, such as self-reported anxiety and depression. The intensity of the child’s problems differed significantly between the group of parents who self-reported anxiety and those who did not. However, no such difference was observed between the group of parents who reported depressive symptoms and those who did not.

The items of the Child Problems subscale mainly reflect the child’s externalized and internalized general behavioral problems. This suggests that these issues could exist prior to the cancer diagnosis and may also be exacerbated following the diagnosis. Even after controlling for medical factors (diagnosis and treatment intensity rated by an oncologist) and sociodemographic factors (age of parents, family status, number of children in family), the relationship between a child’s problems and parental stress reactions remained statistically significant. Our findings on the limited influence of medical and sociodemographic factors confirm the presumption that the best way to predict which parents will have greater or lesser stress due to their child’s illness/treatment is to use a general personality measure (Phipps et al., 2009; Willard et al., 2021).

Although correlation does not allow for the inference of causality (i.e., while a correlation between two variables may be observed, it does not necessarily imply a cause-and-effect relationship between them), we hypothesized that children’s problems, as operationalized in the Child Problems subscale, precede parents’ stress reactions following the diagnosis. In the model proposed here, children’s behavioral and emotional problems may be interpreted as a prognostic factor. This is because the dyadic interrelationship between the child’s externalized and internalized behavioral problems and parental temperamental traits (e.g., neuroticism), which could partially explain the intensity of parental stress reactions after the diagnosis, as well as vulnerability to anxiety and depression, exists prior to the diagnosis. The traumatic experience of cancer treatment may amplify this relationship.

Furthermore, our research gives valuable insights into the structure of cancer-related stress reactions among parents after diagnosis by identifying the main components in which the data varies the most. The primary dimension of parental stress is related to individual discomfort and personal concerns (explaining 27% of the variance in the Stress Reactions subscale), and the secondary one to challenges in parenting a child with cancer and changes in parental roles (19%). This configuration of stress dimensions validates the assumption that parents initially need psychosocial support for themselves in order to care for their children effectively (Kearney et al., 2015) and to reduce their children’s internalized stress symptoms (Fedele et al., 2013).

### 4.4 Illness cognitions as a mediator between children’s problems and parental stress

Multiple linear regression was employed to test the relationship between children’s psychological problems and parental distress symptoms after diagnosis. The study found that both Child’s Problems and Family Beliefs positively predicted Stress Reactions. Causal mediation analysis revealed that Family Beliefs explained 9% of the variance in the relationship between the two. This implies that the impact of a child’s problems on parental stress reactions is partially explained by cancer-related cognitions in the family. This finding underscores the importance of considering the psychological and emotional dynamics within the family unit, along with their cognitions and perceptions of cancer, when addressing the stress reactions of parents in the context of their child’s cancer diagnosis.

This conclusion aligns with existing research that highlights the interconnectedness of family dynamics, parental stress, and the impact of a child’s illness on the family unit. On the other hand, our study offers a modest yet significant contribution to the literature on cancer-related illness cognitions. Along with studies that have established the significant influence of illness beliefs on a patient’s ability to cope with their condition, as well as on their health outcomes and overall quality of life (Ames et al., 2024; Horwood et al., 2024), our findings make a distinctive contribution to the body of literature on cancer-related illness cognitions, shedding light on their mediating role within the dyadic relationship between a child and parent.

Illness cognitions are dynamic predictors of cancer-related outcomes that can be more readily influenced through psychosocial interventions, in contrast to static predictors such as tumor type, physical effects of treatment, previous life events, sociodemographic factors, and personality traits (Bruce, 2006). This study has highlighted the most informative beliefs associated with higher psychosocial risk levels in the family. The results showed significant differences between the Targeted and Universal groups of parents, particularly in their beliefs that *“People will pull away from us”* and *“Cancer is a death sentence.”* These cognitions, along with the belief that the illness would disrupt the family/marriage, were significantly linked to symptoms of parental anxiety and depression, as well as heightened levels of stress reactions following the diagnosis.

The belief that *“Our family will be closer because of this”* was the most informative about parents’ positive adaptation, with a negative correlation between this belief and Family Problems. There was no correlation between parents’ cancer-related cognitions and their perceived support from healthcare teams, extended family, and other social systems. The only exception was the belief that *“My child will be in a lot of pain,”* which correlated negatively with perceived support in parenting.

In conclusion, recognizing the beliefs held by parents regarding their child’s illness and treatment is crucial for tailoring personalized, family-oriented interventions for cognitive restructuring and improving compliance with treatment. The most successful psychological interventions in pediatric oncology target particular elements of the child’s treatment, such as procedural distress, parental reaction to diagnosis, preparation for specific procedures, and long-term psychological consequences. The effectiveness of psychological interventions often relies on a thorough understanding of the thinking patterns of the individuals involved. Therefore, conducting an initial assessment of illness cognitions can assist clinicians in determining the necessary level, intensity, and nature of psychosocial intervention required immediately following a child’s cancer diagnosis.

## 5 Limitations and future research

The relationship found here between children’s problems and parental stress reactions, however, is methodologically challenging. A limitation of this study is its small sample size (*n* = 120), attributed to the relatively low number of pediatric oncology cases in Latvia (50–70 children per year) in proportion to its small population (~1.87 million). The gender imbalance between mothers and fathers accurately reflects the real-world situation, yet it poses a limitation of this study. Nevertheless, a key strength of our research is its inclusion of a significant proportion of the pediatric cancer patient population in Latvia. To our knowledge, no prior research has been conducted on a national sample of pediatric cancer patients in Latvia, making our study crucial for informing future investigations into this population.

Due to the cross-sectional nature of this study and the underpowered sample, it was not feasible to establish the direction of causal relationships between variables, e.g., children’s problems and parental stress reactions. More research using controlled, longitudinal designs and multiple reporters and controlling for children’s problems both before and after diagnosis is needed to delineate this relationship. Nevertheless, our observation offers a significant contribution to the body of pediatric stress research, which has predominantly examined the impact of parental stress on child adjustment difficulties in the opposite direction of the relationship.

Furthermore, the Family Beliefs subscale exhibited a lower level of internal consistency compared to the other subscales. This may affect the reliability of the results and limit their generalizability. The low internal consistency may be attributed to the small sample size, heterogeneity of respondents concerning the time elapsed since diagnosis, ambiguous item phrasing (e.g., “Our family…” instead of “Me…”), as well as the inherent characteristics of these subscales, akin to the psychometric properties observed in the original versions of PAT (Kazak et al., 2018; Pai et al., 2008). Additionally, the measurement of illness cognitions was done only once, shortly after diagnosis, but the sample was not homogenous (8% of patients had a recurrence of a primary tumor and a long treatment experience), therefore it may not have captured changes over time. Research on cancer patients has shown that their understanding of the illness often changes based on internal factors like illness progression and pain, as well as external factors such as information about the illness and medical care. Therefore, it would be beneficial to evaluate changes in illness cognitions at different points during the course of the illness.

The exploration of cultural aspects of pediatric cancer-related cognitions and their mediating role in adjustment is an area that has received fragmental attention in previous research and this study contributes novel insights into pediatric oncopsychology, particularly within the context of Latvia. There is a need for future meta-analytic reviews to provide comprehensive insights that will support cross-cultural cancer communication, reduce parental stress reactions, and improve therapy adherence.

## Data Availability

The raw data supporting the conclusions of this article will be made available by the authors, without undue reservation.
